# Enhanced ADCC Activity of Affinity Maturated and Fc-Engineered Mini-Antibodies Directed against the AML Stem Cell Antigen CD96

**DOI:** 10.1371/journal.pone.0042426

**Published:** 2012-08-03

**Authors:** Sahar Mohseni Nodehi, Roland Repp, Christian Kellner, Joachim Bräutigam, Matthias Staudinger, Natalie Schub, Matthias Peipp, Martin Gramatzki, Andreas Humpe

**Affiliations:** 1 Division of Stem Cell Transplantation and Immunotherapy, Department of Medicine II, Zoological Institute, Christian-Albrechts-University, Kiel, Germany; 2 Department of Structural Biology, Zoological Institute, Christian-Albrechts-University, Kiel, Germany; University of Ottawa, Canada

## Abstract

CD96, a cell surface antigen recently described to be preferentially expressed on acute myeloid leukemia (AML) leukemic stem cells (LSC) may represent an interesting target structure for the development of antibody-based therapeutic approaches. The v-regions from the CD96-specific hybridoma TH-111 were isolated and used to generate a CD96-specific single chain fragment of the variable regions (scFv). An affinity maturated variant resulting in 4-fold enhanced CD96-binding was generated by random mutagenesis and stringent selection using phage display. The affinity maturated scFv CD96-S32F was used to generate bivalent mini-antibodies by genetically fusing an IgG1 wild type Fc region or a variant with enhanced CD16a binding. Antibody dependent cell-mediated cytotoxicity (ADCC) experiments revealed that Fc engineering was essential to trigger significant effector cell-mediated lysis when the wild type scFv was used. The mini-antibody variant generated by fusing the affinity-maturated scFv with the optimized Fc variant demonstrated the highest ADCC activity (2.3-fold enhancement in efficacy). In conclusion, our data provide proof of concept that CD96 could serve as a target structure for effector cell-mediated lysis and demonstrate that both enhancing affinity for CD96 and for CD16a resulted in mini-antibodies with the highest cytolytic potential.

## Introduction

Acute myeloid leukemia (AML) is regarded as a stem cell disorder affecting blood and bone marrow. Although the cancer stem cell model is still under debate, there is strong evidence for the existence of cancer stem cells, especially in AML. AML tumor cells display a clonal hierarchy consisting of a small population of leukemic stem cells (LSCs) with self renewing capacity and their progeny [1], [2], [3]. In contrast to AML blasts, AML-LSCs are able to produce a heterogeneous leukemic xenograft in immunodeficient mice [4]. Due to their slow proliferation and characteristic gene expression profiles AML-LSCs are more resistant to chemotherapeutic agents compared to the more differentiated blasts [5], [6], [7]. Consequently most currently used chemotherapeutic agents kill the majority of AML blasts but are not able to efficiently eliminate AML-LSCs [8]. Therefore, outgrowth of remaining AML-LSCs may eventually lead to relapse of the disease [9]. Targeted therapies directed against AML-LSCs may represent effective therapeutic approaches for AML therapy and, in combination with conventional therapies, may ultimately cure AML patients [8], [10]. For efficient targeting via monoclonal antibodies, identification of cell surface markers preferentially expressed on AML-LSCs but being absent on most non-malignant tissue, especially on normal HSCs, is essential [11]. Several potential target structures on AML-LCSs have been suggested, including CD33 [12], [13], CD44 [14], CD123 [15], [16], CLL-1 [17], [18], CD47 [19] and TIM3 [20]. Recently, expression of CD96 (TACTILE) has been reported on AML-LSCs while only very low expression levels have been found on a small subset of normal HSCs [21].

CD96 is a member of the Ig gene superfamily and its expression has been described on NK-cells and activated T cells [22], [23]. In AML, CD96 expression was detected on the majority of blasts in about 30% of patients [22]. Besides its expression in AML, CD96 was found on a major subset of T-cell acute lymphoblastic leukemias (T-ALL) [22], [24]. The function of CD96 on AML-LSCs or AML blasts is widely unknown. CD96 expressed on NK-cells has been identified as a receptor for CD155 (polio virus receptor) and mediates adhesion of NK-cells to tumor cells, thereby modulating effector function of NK-cells [25].

As demonstrated for other adhesion molecules, CD96 expressed on AML-LSCs may be involved in cell-cell interaction in the bone marrow [26], [27], but further studies are necessary to clarify its role in the pathophysiology of AML. Together, these findings suggest that CD96 may represent a promising target structure for the development of antibody-based therapeutic strategies directed against AML-LSCs. Especially for the treatment of AML, therapeutic antibodies may be used in different clinical settings, including ex vivo purging of AML-LSCs from autologous stem cell grafts or for the targeting of AML-LSCs *in vivo* [21].

Monoclonal antibodies are able to mediate anti-tumor activity by various mechanisms of action [28]. Several findings from mouse models and clinical trials point to an important role for Fc receptors suggesting that indirect effector mechanisms such as antibody dependent cell-mediated cytotoxicity (ADCC) may represent important effector mechanisms *in vivo* [29], [30], [31]. Although therapeutic antibodies were successfully added in many clinical settings there is still room for improvement. Therefore, strategies are developed to increase the antitumor efficacy of monoclonal antibodies by enhancing ADCC activity [32]. Binding affinity to the target antigen as well as Fc binding to activating Fc-receptors has been identified as critical parameters for the ADCC activity of monoclonal antibodies [33], [34]. In addition, the use of recombinant antibody technology may allow design of novel antibody derivatives. Single chain fragment of the variable regions (ScFv) -Fc fusion proteins (mini-antibodies) may represent one promising new molecule format [35], [36]. These molecules display functional characteristics similar to that of complete antibodies but have a molecular weight that is reduced by roughly one third, probably improving tissue or tumor penetration. Recently it has been demonstrated that Fc engineering strategies such as introducing amino acid substitutions [37], [38] or altering the glycosylation profile of antibodies [39], [40], [41], that have been frequently applied to enhance the lytic activity of complete IgG1 antibodies, can also be used to enhance mini-antibody-mediated ADCC [42].

In the present study the V-regions of TH-111, a CD96 mouse monoclonal antibody previously generated in our lab [22], were cloned and used to generate scFv-based mini-antibodies. The effect of affinity maturation and Fc-engineering on the ADCC activity of CD96-directed mini-antibodies was analyzed.

## Materials and Methods

### 1. Bacterial Strains and Constructs

*Escherichia coli* (*E. coli*) Mach1 cells (Invitrogen) were used for the amplification of plasmid DNA. The *E. coli* strain TG1 (Stratagene) and XL1-Blue (Stratagene) were used as host for the preparation of bacteriophages and M13KO7 helper phages (New England Biolabs). The *E. coli* strain BL21 (DE3) (Stratagene) was used for the expression of soluble scFv fragments.

Constructs produced or used in this report are listed in table 1.

**Table 1:** Characteristics of constructs.

Construct name	Specificity	V region	structure	Fc part	Ref.
**ScFv-Fc fusion proteins**					
**CD96-wt-scFv-IgG1-Fc**	CD96	Murine, wildtype,derived from TH-111	scFv-Fc fusion	Wildtype human IgG1	this report
**CD96-S32F-scFv-IgG1-Fc**	CD96	Murine, Affinitymaturated, derivedfrom TH-111	scFv-Fc fusion	Wildtype human IgG1	this report
**CD96-wt-scFv-IgG1-Fc-eng.**	CD96	Murine, wildtype,derived from TH-111	scFv-Fc fusion	Engineered human IgG1 (mutations:S239D/A330L/I332E)	this report
**CD96-S32F-scFv-IgG1-Fc-eng.**	CD96	Murine, Affinitymaturated, derivedfrom TH-111	scFv-Fc fusion	Engineered human IgG1 (mutations:S239D/A330L/I332E)	this report
**CD7-wt-scFv-IgG1-Fc**	CD7	Murine, wildtype,derived from TH-69	scFv-Fc fusion	Wildtype human IgG1	[42]
**CD20-scFv-IgG1-Fc**	CD20	Human, wildtype,derived from clone 7D8	scFv-Fc fusion	Wildtype human IgG1	[42]
**CD20-scFv-IgG1-Fc-eng.**	CD20	Human, wildtype,derived from clone 7D8	scFv-Fc fusion	Engineered human IgG1 (mutations:S239D/A330L/I332E)	[42]
**Monoclonal Abs**					
**TH-111**	CD96		IgG1/kappa	Murine IgG1	[22]
**TH-69**	CD7		IgG1/kappa	Murine IgG1	[24]

### 2. Culture of Eukaryotic Cells

TH-111 hybridoma cells producing a CD96 specific antibody [22], HSB-2 cells (DSMZ; The German Resource Centre for Biological Material) and KG1 cells (DSMZ) were cultivated in RPMI1640-Glutamax-I medium (Invitrogen) supplemented with 10% fetal calf serum (Invitrogen), penicillin and streptomycin (Invitrogen) at concentrations of 100 U/ml and 100 mg/ml, respectively (R10^+^). 293T cells (American Type Culture Collection) were cultivated in DMEM-Glutamax-I medium (Invitrogen) supplemented with 10% fetal calf serum, penicillin and streptomycin at concentrations of 100 U/ml and 100 mg/ml, respectively (D10^+^).

### 3. SDS-PAGE and Western Blot Analysis

Sodium dodecyl sulphate-polyacrylamide gel electrophoresis (SDS-PAGE) was carried out using reducing condition according to standard procedures [43]. The gels were either stained with colloidal Coomassie brilliant blue staining solution (Carl Roth) or were electroblotted to PVDF membrane using standard procedures. For western blot analysis of CD96-scFv-IgG1-Fc and CD96-ECD-IgG1-Fc fusion proteins, the membranes were blocked in 5% NM-TBS (w/v) for 1 h. Membranes were incubated with anti-human IgG HRP-conjugated antibodies (Sigma-Aldrich) diluted in blocking solution (1∶5000) at RT for 1 h. The membranes were washed 3 times with 0.1% Tween 20/TBS (v/v).

For western blot analysis of the E. coli expressed CD96-scFvs, the PVDF membranes were blocked with 3% BSA/PBS (w/v) for 1 h. Then the membrane was incubated with anti-Penta-His antibody (Qiagen, Hilden, Germany) diluted in blocking solution for 1 h. The membrane was washed 3 times for 10 min with washing buffer (TBS buffer supplemented with 0.05% Tween 20 and 0.2% Triton X-100, v/v). The PVDF membranes were incubated with anti-mouse IgG HRP-conjugated antibodies diluted in 10% NM-TBS (w/v) for 1 h. Finally, the membrane was washed 3 times with washing buffer. Blots were developed using SuperSignal West Dura Extended Duration substrate (Thermo scientific) and analyzed using a digital imaging system (Biorad).

### 4. Cloning and Expression of CD96-ECD-IgG_1_-Fc

The extracellular domain of CD96 (CD96-ECD) was cloned and fused to an IgG1 Fc domain to allow purification. Total RNA was prepared from HSB-2 cells using RNeasy Midi kit (Qiagen) according to manufacturer’s instructions. The CD96-ECD was amplified using OneStep RT-PCR kit (Invitrogen) with primers: ECD-For (5′-GATCGCTAGCCACCATGGAGAAAAAATGGAAATACTGTGCTGTC-3′) and ECD-Back (5′-CACAGCGGCCGCCATCTTTGGGCTTATTGACCACAATACC-3′). NheI/NotI digested PCR product was cloned into NheI/NotI digested pSEC-IgG1-Fc resulting in the expression vector, pSEC-CD96-ECD-IgG1-Fc. 293T cells were transiently or stably transfected with pSEC-CD96-ECD-IgG1-Fc as described above (see 2.9). Collected supernatants from transfected cells were applied to protein A affinity chromatography (GE Healthcare) followed by IMAC affinity chromatography (GE Healthcare) according to the manufacturer’s instructions. The final elution peaks were concentrated, extensively dialyzed against PBS and analyzed by SDS-PAGE and capillary electrophoresis to determine amount and purity of the recombinant proteins. To test proper folding of the epitope recognized by TH-111, a capture ELISA assay was established. Briefly, high bind 96-well ELISA plates were coated with either 50 µl of 50 µg/ml CD96 antibody (TH-111) or an irrelevant antibody (TH-69) in PBS at 37°C for 1 h. After discarding the coating solution, blocking was performed with 150 µl of 3% PBA buffer (w/v) at 37°C for 1 h. Blocking solution was replaced by 50 µl of 10 µg/ml CD96-ECD-IgG1-Fc in blocking buffer and incubated at RT for 1 h. The wells were washed 3 times with 150 µl of 1% PBA buffer (w/v) and incubated with polyclonal goat-anti-human IgG HRP conjugated secondary antibodies (Sigma-Aldrich; this secondary antibody is preadsorbed with murine IgG antibodies to prevent unspecific binding to murine IgG immunoglobulins and does not bind to TH-111 (data not shown)). The assay was developed by adding 50 µl of ABTS substrate (Roche Diagnostics) and analyzed in an ELISA reader (TECAN) according to the manufacturers’ instructions.

### 5. Generation of scFv Phage Display Libraries

#### Phage-display library from TH-111 hybridoma cells

Total RNA was prepared from TH-111 hybridoma cells using RNeasy Midi kit (Qiagen) according to the manufacturer’s instructions. 10–15 µg of total RNA was used for first strand cDNA synthesis [44]. PCR amplification of immunoglobulin variable region cDNAs was performed following established procedures [44]. ScFvs were assembled in VL-VH orientation with a 20 amino acid glycine-serine linker by splice overlap extension PCR (SOE-PCR), digested with SfiI, ligated into the phagemid vector pAK100 [44] and used for transformation of XL1-Blue strain of *E. coli* cells. The initial combinatorial scFv library was propagated in 2xTY medium supplemented with 1% glucose and 30 µg/ml chloramphenicol at 37°C under vigorous shaking until the culture density reached an OD_600_ of 0.7. The culture was infected with M13KO7 helper phages (10^12^ pfu) and incubated at 37°C without shaking for 30 min. 200 ml of 2xYT medium supplemented with 1% glucose and 30 µg/ml chloramphenicol was added. Isopropyl β-D-1-thiogalactopyranoside (IPTG) was supplied (final concentration: 0.5 mM). The culture was incubated at 37°C for 1 h under vigorous shaking. Kanamycin was added (final concentration: 25 µg/ml) and the culture was incubated at 30°C overnight in a shaking incubator. The overnight culture was centrifuged and the phages were precipitated by adding ¼ volume of polyethylene glycol (20% PEG 6000/2.5 M NaCl) on ice for 30 min. The phages were subsequently pelleted by centrifugation at 4°C for 20 min and air-dried. The phage pellet was resuspended in 2 ml PBS and was centrifuged at maximum speed for 5 min in a microcentrifuge to remove residual bacterial debris. The phage containing supernatant was stored at 4°C until use.

#### Diversified phage-display library by mutagenesis

Random mutations with low mutation frequency were introduced in the coding sequence of the CD96 scFv by error-prone PCR using GeneMorph II Random Mutagenesis kit (Stratagene) according to manufacturer’s instructions using primers: Mut-Scfor (5′-GATCGGCCCAGCCGGCCATGGCGGACTACAAAGAC-3′) and Mut-Scback (5′-GATCGGCCCCCGAGGCCGCAGAGACAGTGACCAG-3′). PCR conditions were chosen to obtain 1–4 mutations per 1000 base pairs. Amplified scFv was digested with restriction enzyme SfiI, cloned into the phagemid vector pAK100 and used for transformation of XL1-Blue cells. A library with about 1×10^6^ independent clones was generated. Phage preparation was performed as described.

### 6. Panning of the Phage Display Libraries

#### Panning with intact cells

5×10^6^ CD96-positive HSB-2 cells were blocked with 500 µl of 4% non-fat dry milk in PBS (NM-PBS) (w/v) for 30 min under slow agitation. 500 µl of phage suspension was added and incubated at RT for 1.5–2 h. After this incubation step, the HSB-2 cells were washed 10 times with 5 ml of 2% NM-PBS and twice with 5 ml PBS. Bound phages were eluted by incubation with 1.5 ml of 50 mM HCl for 10 min. After neutralization with 500 µl of 1 M Tris at pH 7.5, the cells were pelleted by centrifugation. The supernatant was used for infection of 10 ml exponentially growing *E. coli* TG1 cells at 37°C for 30 min. 20 ml of 2xYT medium supplemented with 1% glucose and 30 µg/ml chloramphenicol was added to the culture and was incubated at 37°C for 2 h under vigorous shaking. After this incubation step, the TG1 cells were super-infected with 1 ml of helper phages (1×10^12^ pfu/ml) at 37°C for 30 min without shaking. 100 ml of 2xYT medium supplemented with 1% glucose and 30 µg/ml chloramphenicol were added and the IPTG concentration was adjusted to 0.5 mM. 1 h after infection, kanamycin was supplied at a final concentration of 25 µg/ml and the culture was allowed to grow overnight at 30°C in a shaking incubator. On the following day, the phages were prepared as described above.

#### Panning of the diversified phage display library with CD96-ECD-IgG1-Fc fusion protein

To allow stringent selection, screening of the diversified library was performed using the purified CD96-ECD-IgG1-Fc protein. Briefly, CD96-ECD-IgG1-Fc (10 µg/ml) was coated to ELISA plates overnight at 4°C. In each round of selection 1×10^12^ bacteriophages were used as input. After 2 h of incubation at RT wells were washed 20 times with PBS supplemented with 1% Triton X-100, 1% Tween 20 (v/v) and 10 times with PBS. (These washing conditions were optimized for complete removal of wild type scFv-expressing bacteriophages). Bound phages were eluted and immediately neutralized as described (see 2.5). Thirty randomly selected clones from the diversified library and from the library after five rounds of screening were sequenced.

### 7. Whole Cell Phage ELISA

1×10^6^ cells per well were blocked with 100 µl of 2% NM-PBS (w/v) in a 96-well plate at RT for 1 h under slow agitation. Cells were pelleted, resuspended in 50 µl phage solution supplemented with 1% non-fat dry milk (w/v). The 96-well plate was incubated at RT for 1–1.5 h under slow agitation. The cells were washed five times with 0.1% NM-PBS (w/v) and twice with PBS. The cell pellets were resuspended in 50 µl of anti-M13 HRP-conjugated antibody (Amersham pharmacia) diluted in 1% NM-PBS (1∶2000) and incubated at RT for 1 h under slow agitation. Cells were washed three times with 0.1% NM-PBS and twice with PBS. Cells were resuspended in 80 µl of ABTS substrate (Roche diagnostics) and the absorbance at 405 nm was scanned in an ELISA reader (TECAN) according to the manufacturers’ instructions.

### 8. Cloning and Bacterial Expression of Soluble scFv Fragments

To allow soluble expression of scFv fragments in E. coli BL21 (DE3), SfiI-digested CD96-scFvs were ligated into SfiI-digested vector pET27b-STREP (unpublished). This vector carries a pelB secretion leader and an N-terminal combined StrepII-6xhistidine tag. The pet27b-STREP-CD96-scFv vectors were propagated in BL21 (DE3) *E. coli* cells. Overnight cultures were diluted in 500 ml 2xYT medium supplemented with 1% glucose and 50 µg/ml kanamycin (OD_600_<0.1). The cultures were grown to an OD_600_ of 0.8–1 at 37°C in a shaking incubator. Protein expression was induced by adding IPTG (final concentration: 0.2 mM) and lowering the temperature to 24°C. After 20 h incubation in a shaking incubator, the *E. coli* cells were collected by centrifugation and periplasmic extracts were prepared using standard protocols [45]. Purification of Strep-tagged CD96-scFv protein was performed on streptactin affinity matrix (Qiagen) using an ÄKTA-purifier system (GE Healthcare) according to the manufacturer’s instructions. The final elution peaks were concentrated and analyzed by SDS-PAGE and capillary electrophoresis to determine amount and purity of the recombinant proteins.

### 9. Cloning and Expression of CD96-mini-antibodies

The CD96-scFvs isolated from the initial phage display library or from the diversified library were digested with SfiI and cloned into SfiI digested pSEC-IgG1-Fc (carrying wild type Fc) or pSEC-IgG1-Fc-eng (carrying an Fc variant optimized for CD16a binding; mutations: S239D/A330L/I332E) [42]; resulting in the expression vectors, pSEC-CD96-wt-scFv-IgG1-Fc, pSEC-CD96-wt-scFv-IgG1-Fc-eng and pSEC-CD96-S32F-scFv-IgG1-Fc-eng. In all constructs the IgG1 allotype background was G1m3. The sequence was confirmed by Sanger sequencing of the final construct.

For transient expression of Fc fusion proteins, 293T cells were transfected with the respective expression vectors using the calcium phosphate method. One day prior to transfection, 3–5×10^6^ cells were seeded in 100 mm tissue culture dishes. The next day, medium was replaced by 8 ml of culture medium. 20 µg of DNA were used for transfection of one 100 mm tissue culture dish according to standard procedures. After 8–10 h incubation at 37°C and 5% CO_2_ in a humidified incubator_,_ the medium was replaced with prewarmed complete culture medium. Supernatant was collected every 24 h for 7 days.

For stable transfection, one day prior to transfection, 5×10^5^ cells were seeded in 3 ml D10^+^ medium in 6-well plates. 2–3 µg of plasmid DNA was used for transfection with Lipofectamine LTX reagent (Invitrogen) according to the manufacturer’s instructions. 48 h after transfection, medium was exchanged and 500 µg/ml hygromycin B (Invitrogen) was applied for the selection of stable clones. Cell lines with stable protein production were identified by limiting dilution and ELISA screening.

Collected supernatants from transient or stably transfected cells were applied to two-step affinity chromatography. In the first step, proteins were enriched using protein A affinity chromatography (GE Healthcare) followed by IMAC affinity chromatography (GE Healthcare) according to the manufacturer’s instructions. The final elution peaks were concentrated, extensively dialyzed against PBS and analyzed by SDS-PAGE and capillary electrophoresis to determine amount and purity of the recombinant proteins.

Gelfiltration chromatography was performed on an ÄKTA purifier (GE Healthcare) using phosphate buffered saline (PBS) as running buffer at a constant flow rate of 0.7 ml/min. 150 µg protein were loaded in a volume of 0.5 ml on a Superdex 200 10/300 GL column (GE Healthcare). Ferritin (440 kDa), human IgG1 (150 kDa), Conalbumin (75 kDa) and Ribonuclease A (13.7 kDa) were used for calibration. Data were analyzed with Unicorn 5.1 software (GE Healthcare, Munich, Germany).

### 10. Flow Cytometry

3×10^5^ cells were incubated with 20 µl of the respective antibody dilution for 30 min on ice. Cells were washed twice with 1% PBA. For CD96-scFvs, the cell pellet was resuspended in 20 µl of 10 µg/ml Penta-His Alexa Fluor conjugate (Qiagen) diluted in 1% PBA. For CD96-scFv-IgG1-Fc fusion proteins, the cell pellet was resuspended either in 20 µl of 10 µg/ml Penta-His Alexa Fluor conjugate or anti-human IgG1-FITC (Beckman-Coulter). The cells were incubated on ice for 30 min. Unbound antibody was removed by washing with PBA. Finally, the cells were resuspended in PFA buffer (1% paraformaldehyde in PBS; w/v) and analyzed on a Cytomics FC500 flow cytometer using the CXP software (Beckman-Coulter). Ten thousand events were collected for each sample using appropriate scatter settings.

For competition binding assays, 5×10^5^ cells were incubated with the antibody-derivative in the presence of a 50-fold molar excess of either TH-111 or an irrelevant monoclonal antibody (TH-69, directed against human CD7) at RT for 30 min. The cells were washed once with PBA and resuspended in 20 µl of polyclonal FITC-conjugated goat-anti-human-IgG1 antibodies diluted in PBA buffer and incubated on ice for 30 min. Unbound antibodies were removed by washing with PBA buffer and the cells were resuspended in PFA buffer and analyzed on a flow cytometer. Ten thousand events were collected for each sample.

### 11. Antibody Dependent Cell-mediated Cytotoxicity (ADCC) Assay

For the isolation of human effector cells, 100 ml of peripheral blood was drawn from healthy volunteers after obtaining written informed consent. The experiments reported here were approved by the Ethics Committee of the Christian-Albrechts-University (Kiel, Germany) in accordance with the Declaration of Helsinki.

Citrate-anticoagulated blood from healthy volunteers was layered over a discontinuous gradient consisting of 70% and 62% Percoll (Biochrom)/Hank’s medium (PAA Laboratories) respectively. After centrifugation at RT for 20 min, MNCs were collected from the serum/Percoll interface. MNCs from healthy volunteers typically contained approx. 60% CD3-positive T-cells, 10–15% CD56-positive NK-cells and 3–10% CD14-expressing monocytes as determined by immunofluorescence staining. Viability of the cells tested by trypan blue exclusion was higher than 95%.

Target cells were labelled with 100 µCi of ^51^Cr per 1×10^6^ cells at 37°C in a humidified CO_2_ incubator for 2 h. After this incubation period, the cells were rinsed three times with R10^+^ medium and incubated with serial dilutions of appropriate antibodies at RT for 30 min. The cells were rinsed once with 10 ml R10^+^ medium and then adjusted to 1×10^5^ cells/ml and added to the 96-well plates (50 µl/well) containing effector cells resulting in a final volume of 200 µl and an E:T ratio of 80∶1. After 3 h incubation at 37°C in the humidified CO_2_ incubator, assays were stopped by centrifugation. 25 µl supernatant from each well was mixed with 150 µl OptiPhase Supermix (Perkin Elmer) in a 96-well flexible plate (Perkin Elmer). The plate was sealed and shaken vigorously for 15 min. ^51^Cr release was counted on a MicroBeta counter and measured in counts per minute (cpm). Maximal ^51^Cr release was determined by adding Triton X-100 (1% final concentration) to target cells and spontaneous release was measured in the absence of effector cells. The percentage of specific lysis of labeled target cells was calculated using the formula:


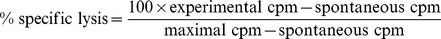


### 12. Homology Modelling of the CD96-scFv

The three-dimensional model of the wild type CD96-specific scFv (CD96-wt-scFv) was calculated using the Rosetta server/algorithm [46]. The surface display model was generated using the Accelrys DS Viewer software (Accelrys, Inc.; San Diego; USA).

### 13. Data Processing and Statistical Analyses

Data are displayed graphically and were statistically analyzed using GraphPad Prism 4.0 software. P-values were calculated using the student’s t-test, one or two-way Anova with Dunn’s or Bonferroni’s post test. The null hypothesis was rejected for p<0.05.

## Results

### 1. Isolation of v-regions from the CD96-specific Hybridoma TH-111

For the generation of human/mouse chimeric mini-antibodies, the functional variable regions from the CD96-specific TH-111 hybridoma were isolated. Hybridoma cell lines may transcribe mRNA species coding for non-functional v-regions [47], [48] therefore a phage display approach was chosen to allow screening for functional v-regions. VL and VH regions were amplified by RT-PCR and amplification products of about 400 bp were derived and fused by SOE-PCR resulting in a PCR-product (scFv) of about 800 bp (data not shown). A corresponding phage display library was generated and subjected to 3 rounds of screening on CD96-positive HSB-2 cells.

**Figure 1:**
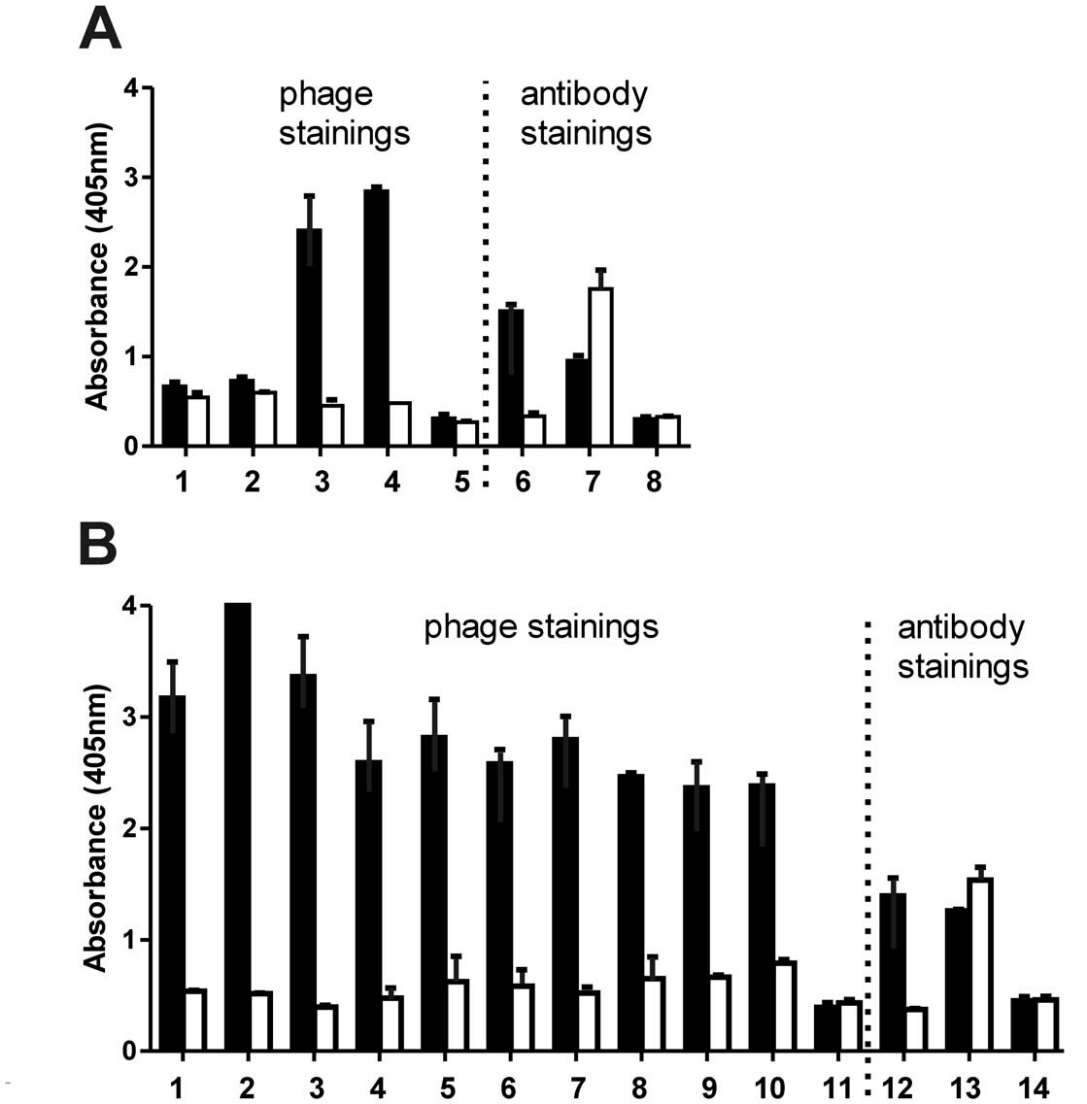
Binding of CD96-phages to CD96-positive HSB-2 cells. **A**) In a polyclonal whole cell-phage ELISA preferential binding of phage preparations to CD96-positive cells was analyzed. HSB-2 (CD96^+^, CD7^+^) and CEM cells (CD96^-^, CD7^+^) were incubated with phages from the original library (1), after the first screening round (2), after the second screening round (3), and after third screening round (4), helper phages (5), TH-111 antibody (6), TH-69 antibody (7), no primary antibody (8) **B**) Whole cell phage ELISA with monoclonal phage preparations. HSB-2 and CEM cells were incubated with phages from 10 single clones (1–10), helper phages (11), TH-111 antibody (12), TH-69 antibody (13), no primary antibody (14). An anti-M13-specific HRP-conjugated antibody was used for detection of phages; HRP-conjugated goat-anti-mouse-IgG antibodies for antibody stainings. □ =  CEM cells; ▪ =  HSB-2 cells.

Phages from the original library, after each screening round and ten randomly selected clones from the last screening round were analyzed by whole cell ELISA (Fig. 1A, B). While the original library demonstrated only low binding activity on HSB-2 cells, strong signals were observed after each screening round, indicating that HSB-2 binding phages were enriched. Phages from screening round two and three as well as the ten clones from the last screening round preferentially bound to HSB-2 cells and demonstrated no or weak binding to CD96-negative CEM cells. Sequencing revealed that the ten single clones harbored identical scFv sequences.

### 2. Construction and Characterization of a CD96-specific Mini-antibody

**Figure 2:**
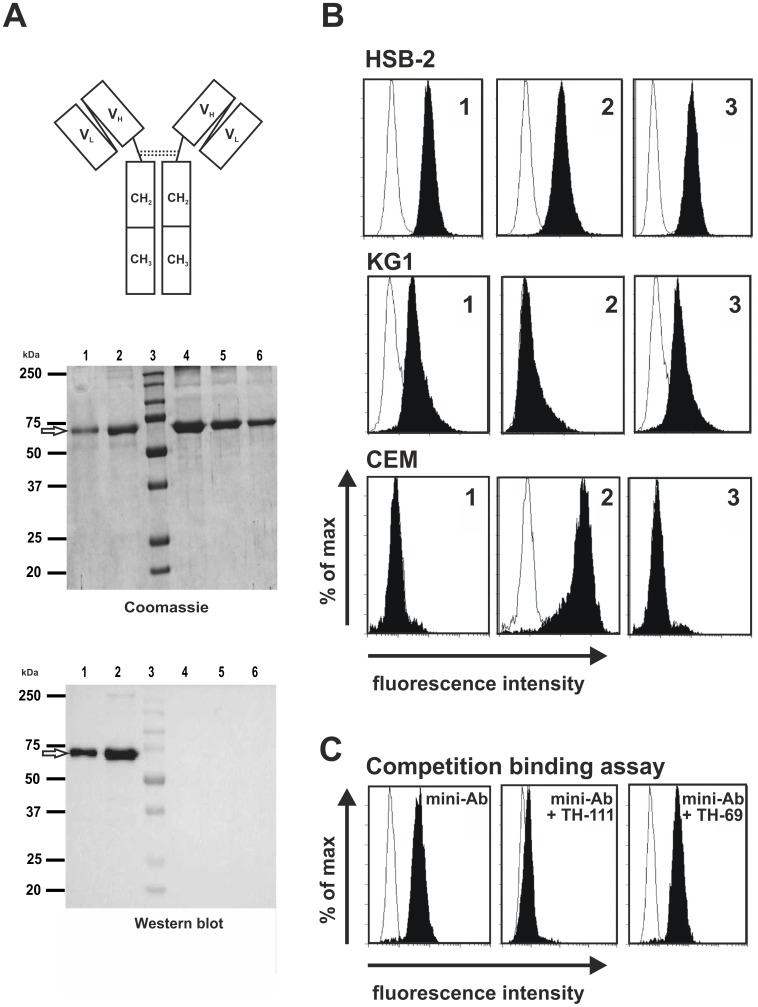
Expression and binding specificity of CD96-wt-scFv-IgG1-Fc (mini-antibody). **A**) Scheme of mini-antibody (upper panel). Coomassie blue stained SDS–PAGE gel (middle panel). Western blot (lower panel). Lanes 1 and 2: purified CD96-wt-scFv-IgG1-Fc protein, lane 3: molecular mass marker, lanes 4–6∶5, 2.5 and 1.25 µg of BSA. Data are representative of at least three experiments that were performed. **B**) Flow cytometric analysis: HSB-2, KG1 or CEM cells were incubated with 1) CD96-scFv-IgG1-Fc, 2) CD7-scFv-IgG1-Fc, and 3) TH-111. irrelevant mini-antibodies served as controls. **C**) Competition binding assay: 1) CD96-wt-scFv-IgG1-Fc, 2) CD96-wt-scFv-IgG1-Fc and TH-111, 3) CD96-wt-scFv-IgG1-Fc and TH-69. Representative experiments are presented.

The selected scFv was converted to a bivalent mini-antibody by genetically fusing to a human IgG1-Fc region containing a modified hinge region as reported earlier (Fig. 2A; [42]). IgG1 was chosen, because this antibody isotype usually triggers several effector functions such as ADCC and CDC considered being important for antitumor activity. The molecules were expressed by secretion in 293T cells. After two-step affinity chromatography, the purified CD96-wt-scFv-IgG1-Fc fusion protein (mini-antibody) was analyzed by SDS-PAGE and Coomassie-staining. One prominent band of about 60 kDa was detected under reducing conditions (Fig. 2A) roughly corresponding to the calculated molecular mass of 56.6 kDa. Under non-reducing condition, CD96-wt-scFv-IgG1-Fc demonstrated a molecular mass of about 110–120 kDa (data not shown), indicating the expected dimer formation of the recombinant molecule. Western blot analysis further confirmed the identity of the purified protein (Fig. 2A).

The novel mini-antibody demonstrated a staining pattern identical to that of TH-111 indicating that the isolated scFv retained the antigen specificity of the parental antibody (Fig. 2B). To further demonstrate that the isolated scFv was CD96 specific and that during the construction process of the scFv fragment the binding specificity of CD96-scFv was not altered, competition binding assays with the parental TH-111 antibody were performed. High concentrations of TH-111 prevented the binding of the CD96 mini-antibody to CD96-positive cells (Fig. 2C) while an irrelevant antibody (TH-69) used at the same concentration did not show this inhibitory effect (Fig. 2C). In conclusion, a functional active CD96-specific scFv and a derived mini-antibody were generated that retained the antigen specificity of the parental murine antibody.

### 3. Construction of CD96-scFv Mutants with Enhanced Binding Affinity

**Figure 3:**
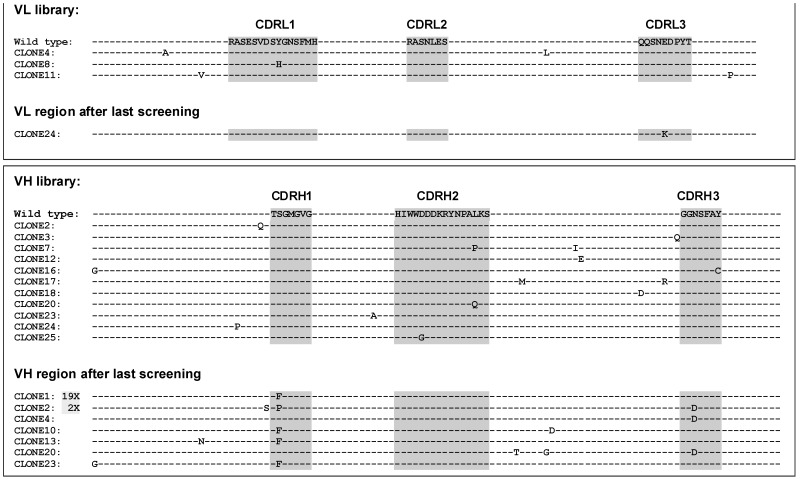
Sequence alignment of the VH and VL domains of mutated CD96-scFv library. After the fifth round of screening, 30 clones were randomly selected and sequences were compared to same number of clones from the original library. The CDR regions are defined according the Kabat numbering scheme [53]. **A**) Light chains from original library and after the fifth round of screening. **B**) Heavy chains from original library and after the fifth round of screening.

Affinity to the target antigen has been demonstrated to critically influence the cytolytic potential of antibodies and antibody derivatives. Therefore, *in vitro* affinity maturation of the scFv fragment by mutagenesis PCR and phage display was performed. The coding sequence of the CD96-specific scFv fragment was amplified using error-prone PCR, thereby introducing random mutations with low frequency. The amplified product was used to generate a “diversified” phage display library with about 1×10^6^ independent clones. To allow stringent screening for enhanced CD96 binding, a recombinant fusion protein composed of the extracellular domain of CD96 and human IgG1-Fc (CD96-ECD-Fc) was produced (data not shown). The diversified library was panned five rounds using immobilized CD96-ECD-Fc as screening antigen. 30 randomly selected phage clones from the last screening round were sequenced and compared to sequences obtained from the original library. At the DNA level 0–4 mutations were introduced in each clone resulting in missense amino acid exchanges in about 1/3 of clones analyzed. Furthermore, mutation sites were randomly distributed in both VH and VL genes (Fig. 3). After the fifth round of screening, 29 of 30 clones carried amino acid exchanges while only one wild type clone was isolated (Fig. 3). These data indicated that phages carrying wild type scFv were successfully eliminated during the selection procedure. Amino acid exchanges were predominantly detected in the complementarity determining region (CDR) 1 domain of the VH domain while only one clone with a mutation in the VL domain was found. The most abundant mutation was detected at amino acid position 32 in the heavy chain which resulted in a serine to phenylalanine substitution (S32F). A missense mutation resulting in a serine to proline exchange (S32P) was the second most abundant mutation at the same amino acid position. Therefore, both mutations resulted in the exchange of the polar serine to a non-polar amino acid. In 5 clones, an amino acid exchange in the CDR3 region of the heavy chain was observed resulting in an asparagine to aspartic acid substitution (N97D).

**Figure 4:**
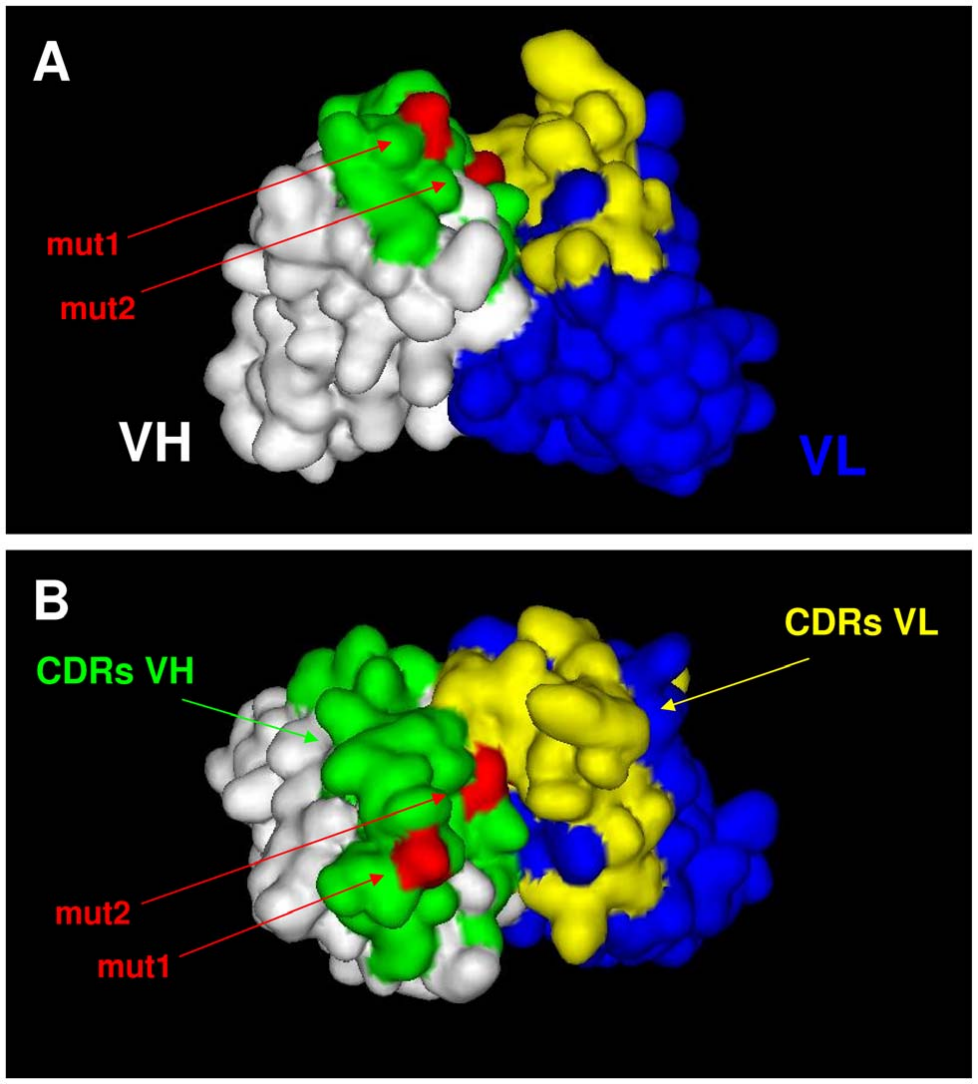
Calculated homology model of wild type CD96-scFv. **A**) A homology model of the wild type CD96-scFv was calculated using the Rosetta Antibody: Fv Homology modelling server [46] and displayed as a surface model using the Accelrys DS Viewer software. **B**) Top view of the model displayed in (A). The 6 loops defining the CDRs were assigned according to the Kabat numbering scheme. Heavy chain CDRs (green), light chain CDRs (yellow), heavy chain frameworks (white), light chain frameworks (blue), positions found mutated after screening (red), mut1 = S32F and mut2 = N97D.

To obtain first indications whether the identified positions were surface exposed in the VH domain and therefore may contribute to CD96 binding by direct interaction, a homology model of the wildtype scFv was calculated (Fig. 4). This model would predict that the two most common positions found to be altered in CDR1 and CDR3 are surface exposed and therefore may directly contribute to CD96 binding. Clone 1 carrying the most abundantly detected mutation S32F was chosen for further analysis.

### 4. Binding Characteristics of Wild Type (CD96-wt-scFv) and Mutated scFv Variant (CD96-S32F-scFv)

To analyze whether the detected amino acid exchanges resulted in altered antigen binding, the CD96-wt- and CD96-S32F-scFvs were produced in *E. coli* in a monovalent format or in 293T cells as bivalent Fc-fusion proteins (an Fc variant optimized for high affinity CD16a binding was used in the described experiments) to allow analysis of avidity effects. The *E. coli* expressed CD96-scFvs demonstrated the expected molecular mass of about 30 kDa while the Fc-fusion proteins displayed a molecular mass of about 60–63 kDa. Western blot analysis confirmed the identity of the purified proteins (data not shown). Dose-dependent binding of CD96-wt-scFv and CD96-S32F-scFv were analyzed by flow cytometry. The CD96-S32F-scFv demonstrated about 4-fold enhanced binding compared to CD96-wt-scFvs (data not shown).

**Figure 5:**
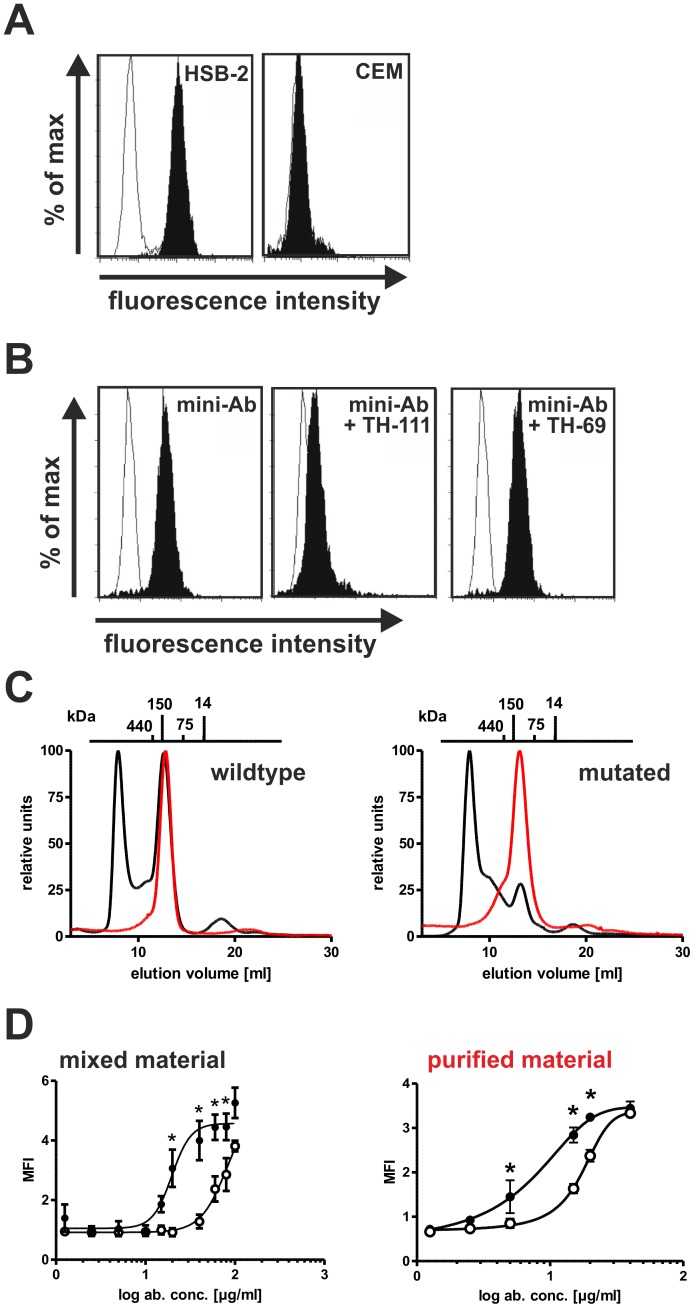
Binding specificity of CD96-S32F-scFv-IgG1-Fc to CD96. **A**) HSB-2 cells or CEM cells were incubated with CD96-S32F-scFv-IgG1-Fc (black) or CD20-scFv-IgG1-Fc (white). **B**) Competition binding assay. Mini-Ab = CD96-S32F-scFv-IgG1-Fc. **C**) Proteins CD96-wt-scFv-IgG1-Fc-eng (left panel) and CD96-S32F-scFv-IgG1-Fc-eng (right panel) were analyzed by size exclusion chromatography directly after affinity chromatography (black lines). Peak fractions containing the proteins with the expected molecular mass were collected and reanalyzed (red lines). **D**) Dose dependent binding of mini-antibodies containing (left panel) or devoid (right panel) of aggregated material was analyzed by flow cytometry using HSB-2 cells. (○): CD96-wt-scFv-IgG1-Fc-eng, (•): CD96-S32F-scFv-IgG1-Fc-eng. Data are presented as mean values +/− SEM from 7 and 3 independent experiments. (*) indicates significant difference in binding between the two binding curves at the indicated antibody concentration (p<0.05).

The affinity maturated CD96-S32F-scFv was further analyzed in the bivalent mini-antibody format. CD96-S32F-scFv-IgG1-Fc-eng bound specifically to CD96-positive HSB-2 cells but lacked binding to CD96-negative CEM cells (Fig. 5A). To further demonstrate that during the affinity maturation process the binding specificity of scFv was not altered, competition binding assays were performed. Binding of CD96-S32F-scFv-IgG1-Fc-eng was almost completely blocked in the presence of molar excess of the parental antibody TH-111 (Fig. 5B).

The bivalent Fc-fusion proteins were further analyzed by size exclusion chromatography. 50–60% of the Fc-fusion protein containing wildtype scFv formed higher molecular mass aggregates. The Fc-fusion protein based on the mutated scFv variant contained even more aggregated material, of up to 75% (Fig. 5 C, black lines). The fractions containing the molecules with the expected molecular mass of 110–125 kDa were collected and reanalyzed (red lines). These data demonstrated that the expected dimeric fraction could be highly enriched using gel filtration chromatography, although the amounts were very low. When dose dependent binding was analyzed, a 4-fold difference in binding was detected compared to wildtype scFv-based mini-antibody when the unfractionated material was used (EC50-values: 19.5 µg/ml (95% CI = 15.7–24.3 µg/ml) and 79.9 µg/ml (95% CI = 32.8–194.7 µg/ml)). Interestingly, when the fractions devoid of aggregates were analyzed a similar difference in binding of 2.5-fold was observed but significantly lower EC50 values were required to achieve maximum binding (EC50-values: 8.33 µg/ml (95% CI = 5.5–12.6 µg/ml) and 18.07 µg/ml (95% CI = 16.6–19.66 µg/ml)). These data suggest that the aggregated material was inactive in antigen binding. Furthermore these data indicated that no avidity effect was observed (Fig. 5D).

### 5. ADCC Activity of Affinity Maturated and Fc-engineered Mini-antibodies

**Figure 6:**
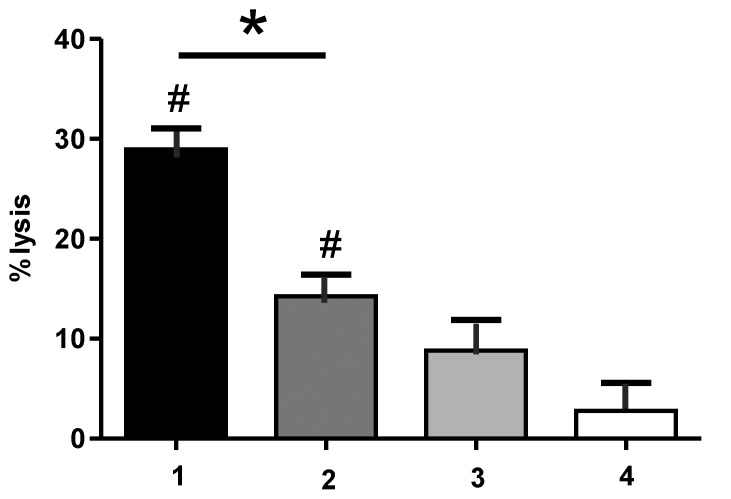
Comparison of ADCC activity at saturating antibody concentrations. ADCC experiments with HSB-2 cells were performed using mononuclear cells as effector cells. (1, **▪**): CD96-S32F-scFv-IgG1-Fc-eng, (2, **▪**): CD96-wt-scFv-IgG1-Fc-eng, (3, **▪**): CD96-wt-scFv-IgG1-Fc, (4, **□**): TH-111. E/T ratio (80∶1). Antibody concentration 200 µg/ml. Data are presented as mean values +/− SEM of 5 independent experiments. (#) extend of lysis significantly different from lysis obtained with the murine TH-111 antibody. (*) extend of lysis significantly different between the two mini-antibodies (p<0.05).

The cytolytic properties of the recombinant CD96 mini-antibodies were investigated by chromium release assay with CD96-positive tumor cells and isolated MNC as effector cells. In a first series of experiments ADCC activity at saturating antibody concentrations was analyzed. Therefore, target cells were incubated with the respective antibodies at concentrations of 200 µg/ml (Note: material containing aggregated material was used in these experiments, because the yield of the mutated variant after gelfiltration was very low) to allow saturated receptor binding. The aggregated material was washed away and the opsonized cells were co-incubated with effector cells. While the murine TH-111 monoclonal antibody triggered very low levels of ADCC (Fig. 6), the optimized mini-antibody variants demonstrated significant cytolytic activity using CD96-positive HSB-2 cells as target cells with CD96-S32F-scFv-IgG_1_-Fc-eng > CD96-wt-scFv-IgG_1_-Fc-eng > CD96-wt-scFv-IgG_1_-Fc ≥ TH-111 (Fig. 6). Irrelevant control mini-antibodies, demonstrated no ADCC activity in the same experimental setting (data not shown). These data indicate that Fc-engineering was essential to trigger significant ADCC using the wild type scFv and that affinity optimization further enhanced the cytolytic capacity.

**Figure 7:**
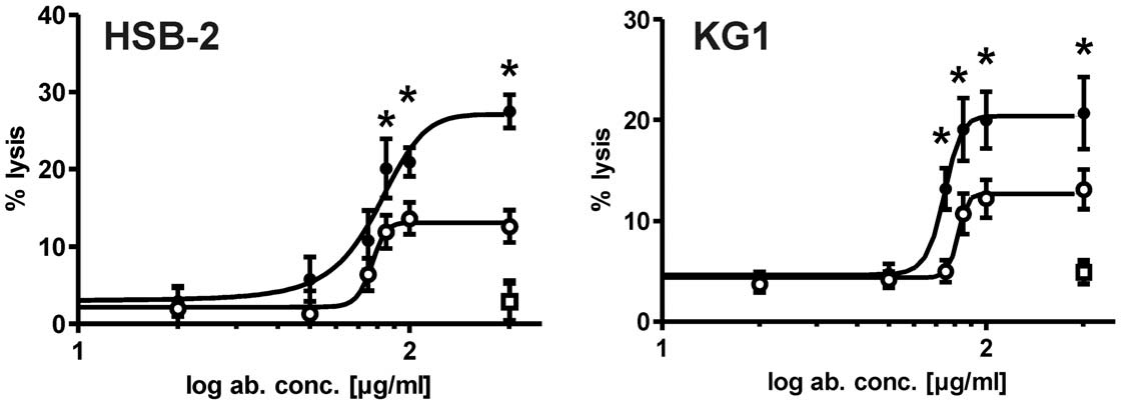
Dose dependent killing of Fc-engineered CD96-mini-antibodies. ADCC with HSB-2 cells. ADCC with KG1 cells. (○): CD96-wt-scFv-IgG1-Fc-eng, (•): CD96-S32F-scFv-IgG1-Fc-eng, (**□**):CD20-scFv-IgG1-Fc-eng, (**•**): TH-111. E/T ratio (80∶1). Data are presented as mean values +/− SEM of 4 and 5 independent experiments. (*) indicates statistically significant difference in lysis between CD96-wt-scFv-IgG1-Fc-eng, CD96-S32F-scFv-IgG1-Fc-eng.

In a last set of experiments, dose-dependent lysis of CD96-positive HSB-2 and KG1 cells was analyzed. The double-engineered CD96 mini-antibody (affinity-maturated scFv and optimized Fc part = CD96-scFv-S32F-Fc-IgG1-eng) showed significantly higher lysis rates compared to its non-affinity maturated counterpart at saturating concentrations, confirming that the gain in affinity translated in improved ADCC potency (Fig. 7). Taken into account that the affinity maturated mini-antibody contained a higher percentage of aggregated material, the observed differences in killing may be more pronounced when purified material is used, but this will require optimization of the production process of that molecule. Differences in the upper plateau may be explained by activation of a higher number of NK cells, thereby increasing the likelihood of a tumor cell to be killed. Alternatively, the higher binding affinity of the engineered antibody variant may allow formation of more tightly stabilized immunological synapses.

## Discussion

The functional v-regions of the CD96-specific hybridoma TH-111 were isolated and used for the generation of a scFv antibody fragment and an affinity maturated variant was isolated. The novel CD96-specific scFvs were used for generating chimeric mini-antibodies that were tested for their capacity to trigger effector cell-mediated killing of opsonised target cells. Both, Fc-engineering and affinity maturation were demonstrated to enhance ADCC activity of the derived CD96-specific mini-antibodies.

Although progress has been made in the treatment of AML patients, still the current five-year survival rate of patients who receive intensive chemotherapy is in the range of 30–40%. For older patients (>60 years) the situation is even worse with a five-year survival rate of about 15%. Therefore novel treatment options are urgently needed. Targeting AML leukemic stem cells may therefore represent an attractive approach, but to date suitable target structures are rare. CD96 may represent a promising candidate with a quite restricted expression profile compared to other suggested target antigens on AML-LSC. Similar to other tumor associated antigens, CD96 is not exclusively expressed on AML leukemic stem cells. Importantly CD96 is not present on normal hematopoietic stem cells. In the hematopoietic system CD96 expression is found at very low levels on T cells and NK cells ([22], [23]). In addition, moderate expression has been found in non-hematopoietic tissues, e.g. on the mucosal epithelium of the small and large intestine and on vascular endothelium. Similar to other antigens (CD33, Her2, EGFR) which are used as therapeutic targets and that are not tumor cell-specific, first in men studies with novel drugs targeting such new target structures have to be carefully performed, and it has to be determined whether a safe therapeutic window exists. Besides targeting AML LSC in vivo, CD96 may also represent an interesting target structure for ex vivo purging of autologous stem cell grafts. In this setting the expression profile does not compromise therapeutic application.

In the past, well characterized murine hybridoma lines served as starting points for the development of antibody-based therapeutics. Although technologies are available to generate fully human antibodies or to humanize non-human v-regions, especially chimeric antibodies have proven their therapeutic potential in a variety of clinical settings. In case that chimerization of the antibody constructs as described here is not sufficient to prevent an immune response when clinically applied, techniques are available to humanize the murine v-regions. Various strategies have been described, such as CDR-grafting, specificity-determining residue (SDR) grafting, human string content optimization, framework shuffling and phage display approaches (e.g. guided selection), which in theory should further reduce potential immunogenic epitopes. Isolation of the functional v-regions represents the first step in generating chimeric or humanized antibody-derivatives. Several approaches have been pursued in isolating v-regions from hybridoma cell lines [44], [48], [49]. While hybridoma cell lines in general express one monoclonal antibody at the protein level, several reports described hybridoma lines that predominantly expressed mRNA species coding non-functional v-regions compromising isolation of the coding sequences of the functional v-regions [47], [48]. A phage display approach was chosen to isolate the functional v-regions from the TH-111 hybridoma. Interestingly, the original phage display library demonstrated only very weak binding activity to CD96-positive tumor cells, indicating that an excess of non-functional scFvs were present in the original library (Fig. 1). After three rounds of selection all tested single clones preferentially bound to CD96-positive tumor cells and harboured identical scFvs, indicating that functional v-regions were efficiently enriched.

Fc receptor mediated effector mechanisms such as ADCC have been demonstrated to be important for the in vivo activity of therapeutic antibodies [30]. Therefore, optimizing selected antibody features in order to enhance ADCC may be beneficial for potential therapeutic application. Affinity and avidity to the target antigen have been demonstrated to impact the cytotoxic potential of antibody derivatives [34], [50], [51]. Especially indirect effector mechanisms such as ADCC are critically affected by the affinity to the target antigen. Therefore, the CD96-specific scFv was subjected to in vitro affinity maturation. While the randomly introduced mutations in the diversified library were roughly equally distributed over the complete scFv sequence, after five rounds of stringent selection mutations were predominantly detected in the CDR1 and CDR3 regions of the heavy chain. Only 1 out of 30 analyzed clones had an amino acid exchange in the VL domain. These data indicate that mutations in the VL region resulted in scFv variants with diminished or similar binding characteristics in the range of the wild type scFv and were therefore eliminated during the stringent screening process. Homology modelling of the wild type scFv suggested that the two most common mutated positions in the CDR1 and CDR3 region of the heavy chain are surface exposed and consequently may represent direct interaction sites of antibody and antigen. The most common mutation isolated (S32F) resulted in a substitution of serine to phenylalanine, a polar amino acid vs a non-polar amino acid. In accordance, it has been demonstrated that hydrophobic interactions frequently are the major driving forces for protein-protein interactions [52]. The single amino acid exchange resulted in a 4-fold increase in binding affinity when monomeric scFv fragments were analyzed. Interestingly no “avidity effect” was observed when divalent antibody derivatives were analyzed (Fig. 5). This may be explained by the low antigen density of CD96 detected on most CD96-positive cell lines.

Besides affinity to the target antigen also affinity to activating Fc receptors has been demonstrated to impact ADCC activity. Different approaches are actively developed to optimize therapeutic antibodies by Fc engineering [32]. Recently, we have demonstrated that besides glyco-engineering also protein-engineered Fc variants enhance the cytotoxic capacity of scFv-based mini-antibodies [42]. In the current report wild type human IgG1-Fc and a human IgG1-Fc variant optimized for CD16a-binding [37] was used for the generation of mini-antibodies. In ADCC experiments at high antibody concentrations significant lytic activity with wild type scFv was only observed when the Fc-engineered Fc variant was used. Importantly, killing activity of the Fc-engineered mini-antibody was further enhanced when it was used with the affinity maturated scFv. This is in accordance with other reports demonstrating that ADCC activity correlates with antibody affinity [34] **.** In conclusion, our data provide proof of concept that ADCC, an important effector mechanism of therapeutic antibodies, can be efficiently triggered via CD96-specific antibodies. Further experiments have to demonstrate whether the described molecules are able to eradicate AML leukemic stem cells in the clinical setting.
